# 

**Published:** 1889-09

**Authors:** 


### CONTENTS.

COMMUNICATIONS.

The Relation of the Teeth to the Diseases of the General System..... 421
The Third Molar, Wisdom Teeth, or Dens Sapientiæ.................... 430
Fractured Jaw.......................................................... 439

PROCEEDINGS.

The Iowa State Dental Society.............................................. 442
Excerpts................................................................ 450

SELECTIONS.

Physical Education....................................................... 459
Ice Water.................................................................. 462
Phenacetin in the Treatment of Neuralgia.............................. 463
Hare-Lip and Cleft Palate.................................................. 464
Abortive Treatment of Whitlow............................................ 465

EDITORIAL.

American Dental Association.............................................. 466
The Association of Dental Faculties...................................... 467
Dr. Wm. H. Dwinelle—Fifty Years........................................ 468
A Crown and Bridge Case, 1859............................................ 469
Medical and Dental Department, Central University of Kentucky... 470
Transactions.............................................................. 471
Hayden Dental Society of Chicago......................................... 471
Society Meeting—Seventh Ohio District.................................... 471

